# Prognostic value of human leukocyte antigen G expression in solid tumors: a systematic review and meta-analysis

**DOI:** 10.3389/fimmu.2023.1165813

**Published:** 2023-05-18

**Authors:** Jorge Bartolome, Consolacion Molto, Javier David Benitez-Fuentes, Gonzalo Fernandez-Hinojal, Aranzazu Manzano, Pedro Perez-Segura, Abhenil Mittal, Faris Tamimi, Eitan Amir, Alberto Ocana

**Affiliations:** ^1^ Experimental Therapeutics Unit, Department of Medical Oncology, Hospital Clinico San Carlos and Health Research Institute of the Hospital Clinico San Carlos (IdISSC), Madrid, Spain; ^2^ Division of Medical Oncology and Hematology, Department of Medicine, Princess Margaret Cancer Centre and University of Toronto, Toronto, ON, Canada; ^3^ Department of Clinical Oncology, Velindre Cancer Centre, Cardiff, United Kingdom; ^4^ Department of Medical Oncology, Clinica Universidad de Navarra, Madrid, Spain

**Keywords:** tumors, HLA-G, human leukocyte antigen G, meta – analysis, cancer

## Abstract

**Introduction:**

Identification of modulators of the immune response with inhibitory properties that could be susceptible for therapeutic intervention is a key goal in cancer research. An example is the human leukocyte antigen G (HLA-G), a nonclassical major histocompatibility complex (MHC) class I molecule, involved in cancer progression.

**Methods:**

In this article we performed a systematic review and meta-analysis on the association between HLA-G expression and outcome in solid tumors. This study was performed in accordance with PRISMA guidelines and registered in PROSPERO.

**Results:**

A total of 25 studies met the inclusion criteria. These studies comprised data from 4871 patients reporting overall survival (OS), and 961 patients, reporting disease free survival (DFS). HLA-G expression was associated with worse OS (HR 2.09, 95% CI = 1.67 to 2.63; P < .001), that was higher in gastric (HR = 3.40; 95% CI = 1.64 to 7.03), pancreatic (HR = 1.72; 95% CI = 0.79 to 3.74) and colorectal (HR = 1.55; 95% CI = 1.16 to 2.07) cancer. No significant differences were observed between the most commonly utilized antibody (4H84) and other methods of detection. HLA-G expression was associated with DFS which approached but did not meet statistical significance.

**Discussion:**

In summary, we describe the first meta-analysis associating HLA-G expression and worse survival in a variety of solid tumors.

**Systematic Review Registration:**

https://www.crd.york.ac.uk/PROSPERO/, identifier CRD42022311973.

## Introduction

Identification of modulators of the immune response with inhibitory properties that could be susceptible for therapeutic intervention is a key goal in cancer research. The human leukocyte antigen G (HLA-G) is a nonclassical major histocompatibility complex (MHC) class I molecule that belongs to the group of HLA-class Ib (1). HLA-G binds to the leukocyte Ig-like receptor subfamily B member 1 (LILRB1) and member 2 (LILRB2), and the killer immunoglobulin-like receptor 2DL4 (KIR2DL4) (2, 3). This molecule induces tolerance towards both innate and acquired immune cells. HLA-G has been associated with critical functions in maternal tolerance of the fetus during pregnancy, and its presence at this immunologically privileged site was proposed as a mechanism used by the fetus to avoid rejection by the mother’s immune system (1–5).

In recent years, the distribution of HLA-G in normal tissues has been found to be broader than initially thought. HLA-G molecules have been detected in embryonic (amniotic cells and fluid, endothelial cells from the chorionic villi and erythroid cells) and in some adult tissues (thymic epithelial cells or the bone marrow in cells of the erythropoietic lineage) (4).

This immune-suppressive function supports a role of HLA-G in tumor development and progression. In malignancy, pathologic HLA-G expression was first found in melanoma samples with no expression in adjacent normal tissues (6). Since this report, HLA-G expression has been associated with various malignancies such as breast cancer, colorectal cancer, cervical cancer, endometrial carcinoma, oesophageal cancer, Ewing sarcoma, gastric cancer, and lung cancer, among others (7). HLA-G expression in solid tumors has been associated with advanced disease stage, tumor metastasis, poor prognosis or poorer disease-free survival (7–10).

Recently agents targeting HLA-G or their receptors, such as the LILRB family of proteins have shown preclinical activity, and some of these agents have transitioned to clinical testing (11, 12). These drugs predominantly act on myeloid derived suppressor cells (13). Given the differential immune profile at different stages of cancer progression we aimed to understand the association between HLA-G expression and clinical parameters and with patient outcome.

In this systematic review, we aimed to explore the prognostic association between HLA-G in solid tumors with a particular focus on early-stage disease where the immune microenvironment plays a central role. We hypothesised that the expression of HLA-G would be associated with worse outcomes due to its inhibitory effects on the immune response.

## Methods

### Study design

This systematic review and meta-analysis were performed in accordance with the Preferred Reporting Items for Systematic Reviews and Meta-Analyses (PRISMA) and was conducted following the Cochrane Handbook for Systematic Reviews of Interventions recommendations. This study was registered in PROSPERO (registration number: CRD42022311973).

### Search strategy and selection criteria

We searched MEDLINE (host: PubMed) and EMBASE to identify studies published from inception to April 24, 2022, and which evaluated: 1) HLA-G expression in solid tumors; 2) reporting hazard ratio (HR) and 95% confidence interval (CI) or a *P* value for overall survival (OS). In a secondary analysis, studies providing a HR for disease-free survival (DFS) were included. Only studies reporting on multivariable Cox proportional-hazards regression analyses were comprised. Those reporting univariable analyses were excluded. The titles identified by the initial search were evaluated, and potentially relevant publications were retrieved in full. Three authors (JBA, JDBF and GFH) reviewed the full articles for eligibility independently. Discrepancies were resolved by consensus. The following MeSH terms were used for the search: “neoplasm”, “malignancy”, “tumor”, “carcinoma”, “cancer” “human leukocyte antigen G” and “HLA-G”. We focused our search on studies performed in adults and reported in English. Non-human studies or preclinical studies, non-malignant pathology, haematological malignancies, case reports, literature reviews, letters to editors and studies with no survival data as specified in the eligibility criteria were excluded. With the emphasis being the association between HLA-G expression and outcomes in early-stage/curable malignancies, studies with more than 50% of patients in the metastatic setting were also excluded.

### Data extraction

Data were collected by one reviewer (CMV) and quality assessment was conducted by a second author (EA). Discrepancies were resolved by consensus. All data were extracted from primary publications and their associated online appendices. Data were collected into an electronic database, which included the following data: study summary characteristics such as tumor type, setting (curative *vs* palliative), number of patients, proportion of patients with advanced and early stage disease, mean age, gender, median follow-up (months), type of HLA-G detection (tissue *vs* plasma), detection technique (Immunohistochemistry [IHC], Enzyme-Linked Immunosorbent Assay [ELISA] or mRNA), antibody used for detection (when appropriate) and, proportion of patients with HLA-G expression. We also extracted outcome data such as OS and DFS from multivariable Cox proportional-hazards regression analyses.

### Statistical analysis

Extracted data were combined into a meta-analysis using Review Manager v5.4 analysis software (Cochrane Collaboration, Copenhagen, Denmark). In light of the expectation for substantial clinical heterogeneity, estimates of HRs were weighted and pooled using the generic inverse-variance and random-effect model (14, 15) irrespective of statistical heterogeneity. A *post-hoc* sensitivity analysis was also performed to exclude any studies in which HRs were not adjusted for age in multivariable analyses. Meta-regression was performed using SPSS version 28.0 (IBM Corp, Armonk, NY, USA) and comprised linear regression weighted by inverse-variance using the weighted least squares (mixed effect) function (16). The following variables were regressed against the natural logarithm for the HR for OS: mean age, proportion of females, proportion of patients with advanced stage, median follow-up (months) and proportion of HLA-G positive. For meta-analysis, statistical significance was defined as *P <*0.05. For meta-regression, in light of the small number of studies resulting in low statistical power, significance was defined quantitatively using thresholds defined by Burnand et al. (17) (quantitative significance defined as ß coefficient >0.28). Publication bias was explored by visual inspection of the funnel plots. No corrections were applied for multiple significance testing.

## Results

### Study characteristics

**Figure 1:**
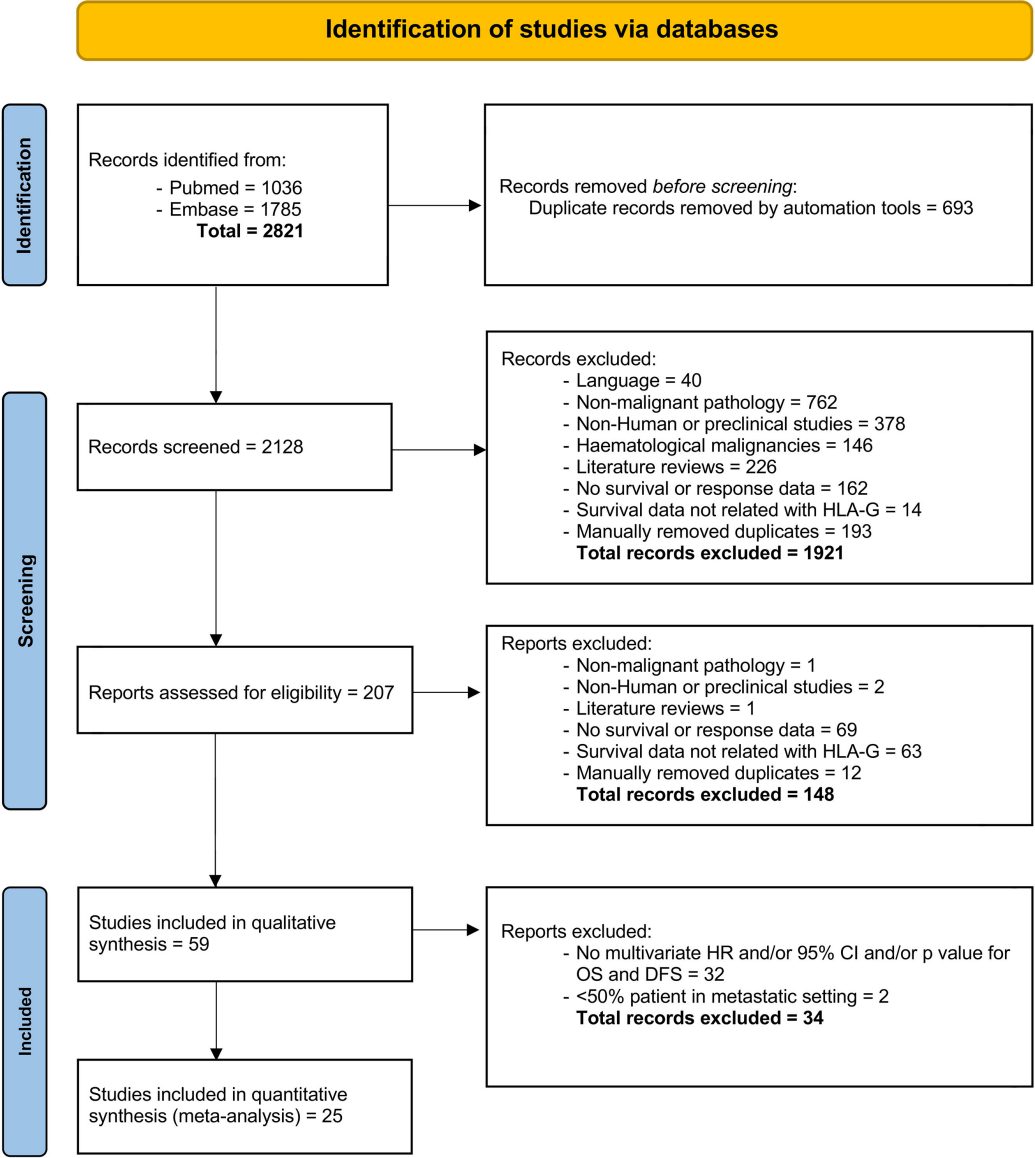
PRISMA flow chart of the study selection process.

Of 2821 studies identified in the initial search, after removal duplications, 2128 studies were screened by title and abstract, with 207 reports were assessed for eligibility, including full text. Finally, a total of 25 studies (10, 18–40) met the inclusion criteria (see **Figure 1** for study selection schema). Of these studies, 25 studies comprising data from 4871 patients reported OS outcomes and among them, only 5 studies (961 patients) reported DFS data in addition to OS. The characteristics of the included studies are shown in **Tables 1**, **2**. Overall, 20 (80%) comprised studies with a mixed group of patients treated in the curative and palliative setting, 21 (84%) used tissue for HLA-G detection and 14 (56%) used anti-HLA-G antibody, clone 4H84. **Supplementary Table 1** provides detailed information about the histological type, stage and the definition utilized for HLA-G expression positivity.

**Table 1:** Characteristics of included studies.

Studies (N = 25)	Patients (N)	Disease site	Setting	Female (%)	Mean age (years)	Median follow-up (months)
Bennedsen (18)	188	Colorectal Cancer	Curative and Palliative	53	71	42
Boujelbene (19)	61	Vulvar Cancer	Curative and Palliative	100	66	NA
Cai (20)	173	Hepatocarcinoma	Curative and Palliative	NA	NA	37
Du (21)	179	Gastric Cancer	Curative and Palliative	27	NA	21
Feiyan Jiao (22)	1037	Colorectal Cancer	Curative and Palliative	58	62	60
Hiraoka (23)	98	Pancreatic Cancer	Curative and Palliative	37	65	17.6
Jung (24)	41	Ovarian Cancer	Curative and Palliative	100	52	NA
König (25)	190	Breast Cancer	Curative	100	51	53
Li (26)	178	Colorectal Cancer	Curative and Palliative	44	65	47
Lin (27)	79	Esophageal Cancer	Curative and Palliative	29	58	36
Murdaca (28)	94	Gastric Cancer	Curative	60	71	61
Reimers (29)	484	Colorectal Cancer	Curative and Palliative	64	65	NA
Samadi (30)	100	Colorectal Cancer	Curative and Palliative	41	51	NA
Schutt (31)	137	Lung Cancer	Curative and Palliative	31	69	9
Sideras (32)	224	Pancreatic Cancer	Curative	41	67	NA
Wan (33)	49	Gastric Cancer	Curative and Palliative	33	60	NA
Wang (34)	212	Hepatocarcinoma	Curative and Palliative	14	60	NA
Wook (24)	41	Ovarian Cancer	Curative and Palliative	100	52	NA
Xu (35)	122	Pancreatic Cancer	Curative	42	62	13.5
Ye (36)	201	Colorectal Cancer	Curative and Palliative	47	64	27
Yie (10)	121	Esophageal Cancer	Curative and Palliative	18	58	36
Yie (37)	160	Gastric Cancer	Curative and Palliative	28	63	36
Zhang (38)	457	Colorectal Cancer	Curative and Palliative	42	66	46.5
Zhang-Yan Guo (39)	102	Colorectal Cancer	Curative and Palliative	59	NA	NA
Zhou (40)	143	Pancreatic Cancer	Curative	39	62	13

NA, Not available.

**Table 2:** HLA-G characteristics and reported outcomes of included studies.

Studies (N = 25)	HLA-G positive (%)	HLA-G detection type	HLA-G detection technique	HLA-G detection antibody	Reported endpoints
Bennedsen (18)	9	Tissue	IHC	4H84	OS and DFS
Boujelbene (19)	20	Tissue	IHC	4H84	OS and DFS
Cai (20)	57	Tissue	IHC	MEM-G/1	OS
Du (21)	50	Tissue	IHC	4H84	OS and DFS
Feiyan Jiao (22)	40	Plasma	ELISA	–	OS
Hiraoka (23)	37	Tissue	IHC	4H84	OS
Jung (24)	37	Tissue	IHC	4H84	OS
König (25)	13	Plasma	ELISA	–	OS
Li (26)	50	Plasma	ELISA	–	OS
Lin (27)	66	Tissue	IHC	4H84	OS
Murdaca (28)	26	Tissue	IHC	4H84	OS
Reimers (29)	28	Tissue	IHC	4H84	OS and DFS
Samadi (30)	25	Tissue	IHC	4H84	OS
Schutt (31)	40	Plasma	ELISA	–	OS
Sideras (32)	15	Tissue	IHC	4H84	OS
Wan (33)	61	Tissue	IHC	4H84	OS and DFS
Wang (34)	50	Tissue	mRNA	–	OS
Wook (24)	NA	Tissue	IHC	4H84	OS
Xu (35)	64	Tissue	IHC	4H84	OS
Ye (36)	67	Tissue	IHC	HGY	OS
Yie (10)	91	Tissue	IHC	HGY	OS
Yie (37)	71	Tissue	IHC	4H84 and HGY	OS
Zhang (38)	60	Tissue	IHC	4H84	OS
Zhang-Yan Guo (39)	71	Tissue	IHC	MEM-G/2	OS
Zhou (40)	14	Tissue	IHC	4H84 and CD3	OS

HLA-G, Human Leukocyte Antigen G; IHC, Immunohistochemistry; ELISA, Enzyme Linked Immunosorbent Assay; OS, Overall Survival; DFS, Disease Free Survival; NA, Not available.

### Overall survival

**Figure 2:**
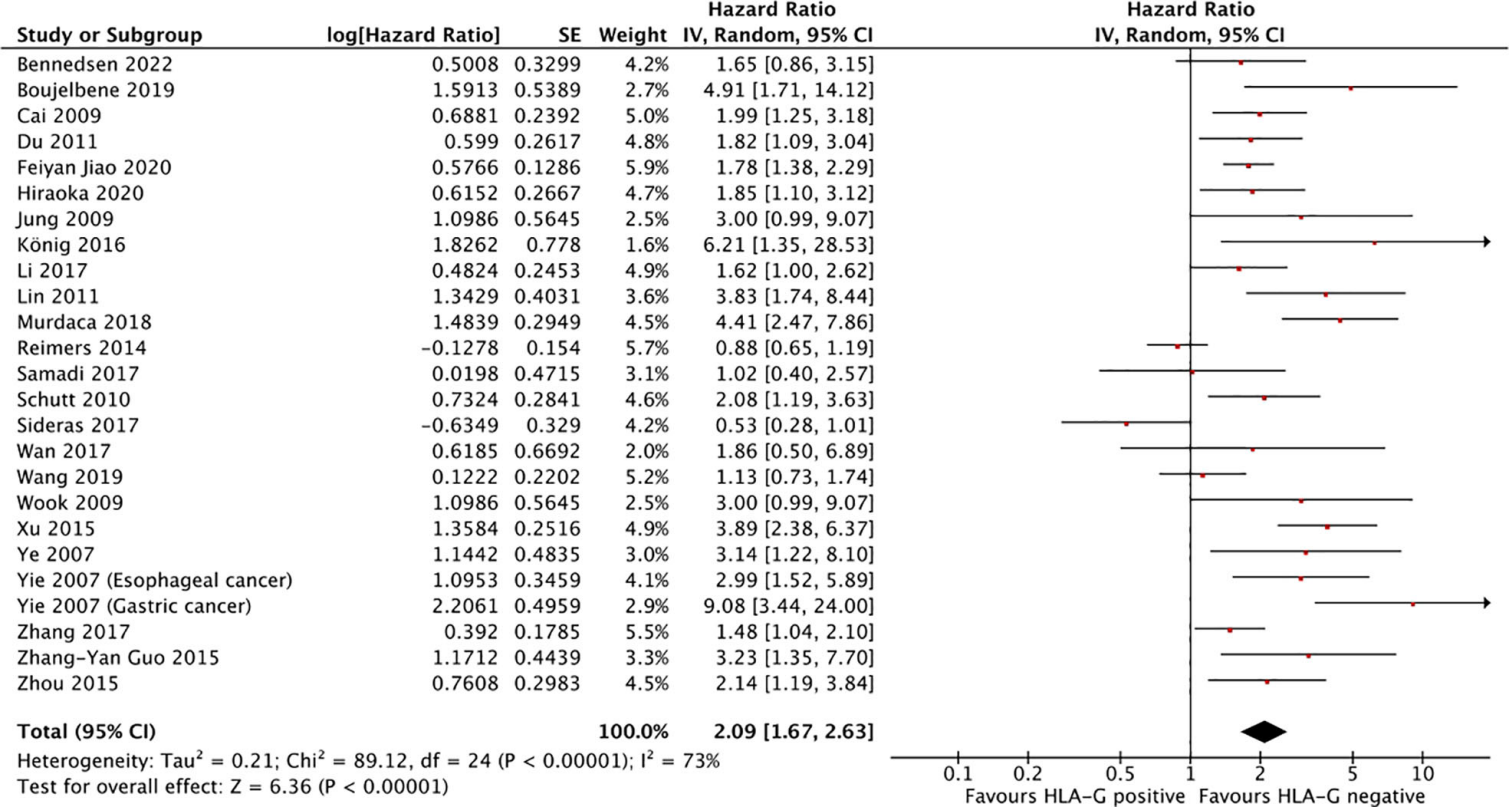
Forest plot showing hazard ratio for OS for HLA-G positive. Hazard ratios for each study are represented by the squares, the size of the square represents the weight of the study in the meta-analysis, and the horizontal line crossing the square represents the 95% confidence interval (CI). All statistical tests were two-sided.

**Figure 3:**
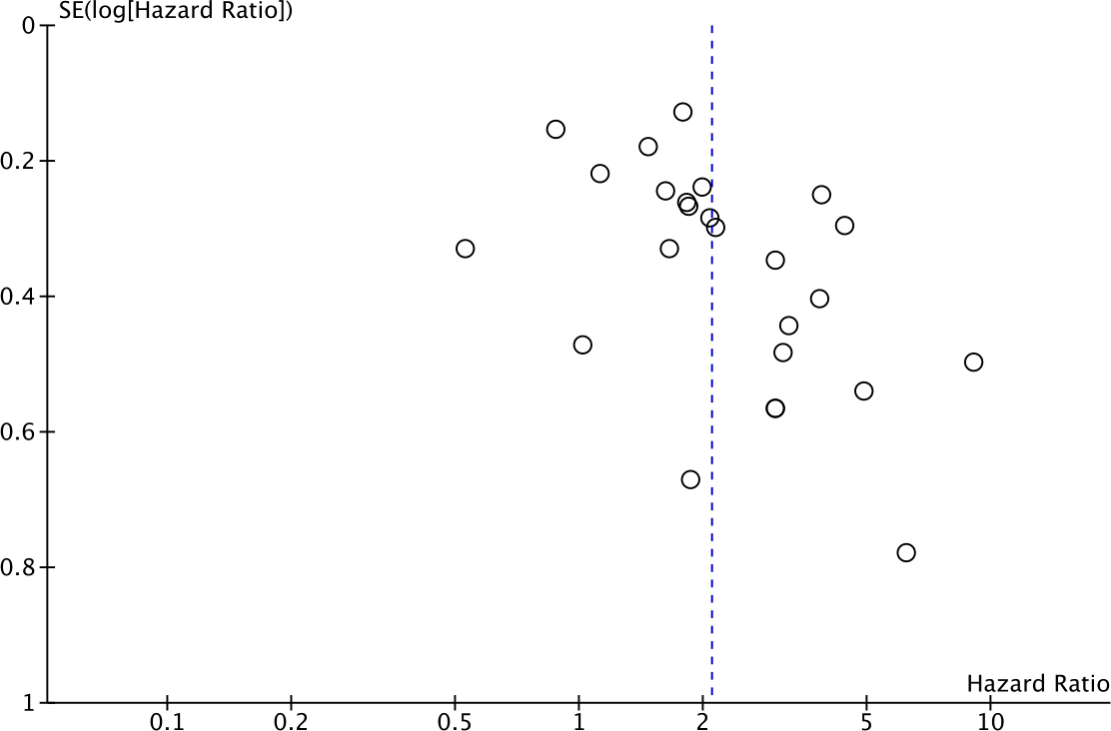
Funnel plot of hazard ratio for OS for HLA-G positive (horizontal axis) and the standard error (SE) for the hazard ratio (vertical axis). Each study is represented by one circle. The vertical line represents the pooled effect estimate.

Twenty-five studies comprising 4871 patients reported HRs for OS. Seven of the eligible 25 studies (28%) reported a non-statistically significant HR (i.e., the 95% confidence intervals crossed 1); a forest plot of all studies is presented as **Figure 2**. Overall, HLA-G expression was associated with worse OS (HR 2.09, 95% CI = 1.67 to 2.63; P <.001). The funnel plot of HR for OS for HLA-G expression is shown in **Figure 3**. Visual inspection suggested potential publication bias with fewer studies with higher error (typically smaller sample size) showing a protective effect from HLA-G expression.

**Figure 4:**
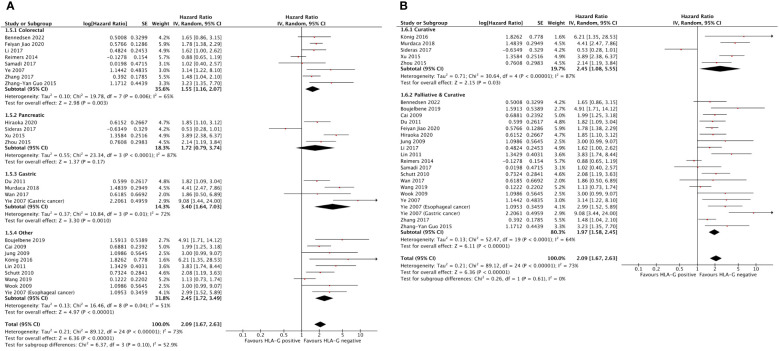
Forest plots showing hazard ratio for OS for HLA-G positive. **(A)** Hazard ratios by disease subgroups. **(B)** Hazard ratios by disease setting. Hazard ratios for each study are represented by the squares, the size of the square represents the weight of the study in the meta-analysis, and the horizontal line crossing the square represents the 95% confidence interval (CI). All statistical tests were two-sided.

The effect of HLA-G on OS among disease site subgroups is shown in **Figure 4A**. The prognostic effect of HLA-G was highest in gastric cancer (HR = 3.40; 95% CI = 1.64 to 7.03), followed by pancreatic cancer (HR = 1.72; 95% CI = 0.79 to 3.74) and colorectal cancer (HR = 1.55; 95% CI = 1.16 to 2.07). The hazard ratio for the subgroup of other unselected solid tumors was 2.45 (95% CI = 1.72 to 3.49). Differences between disease subgroups approached but did not meet statistical significance (P for subgroup difference = .10).

The effect of HLA-G on OS among disease setting is shown in **Figure 4B**. The hazard ratios were 2.45 (95% CI = 1.08 to 5.55) for nonmetastatic disease and 1.97 (95% CI = 1.58 to 2.45) for a mixed group consisting of studies that included both metastatic and nonmetastatic patients. Although the effect of HLA-G expression in nonmetastatic disease was numerically higher value than in mixed metastatic and nonmetastatic disease, this difference was not statistically significant (P for subgroup difference = .61).

In the *post-hoc* sensitivity analysis, 3 studies (Cai et al, 2019; Du et al, 2011; Zhang-Yan Guo et al, 2015) (20, 21, 39) were excluded as they did not adjust for age in the multivariable model. Results showed an unchanged effect size (HR 2.10, 95% CI 1.62 to 2.71, p<.001).

The effect of HLA-G on OS among detection type, detection technique and antibody used are presented as **Supplementary Figure 1** (available online). There was neither a magnitude of effect nor statistically significant difference between the most commonly utilized antibody (4H84) and other methods of detection of HLA-G expression.

Meta-regression analysis is presented in **Table 3**. Overall, there was a quantitatively significant, but non-statistically significant negative association between age and worse OS with HLA-G expression (β = 0.314; P = .155).

**Table 3:** Meta-regression OS Analysis.

Variables	Coefficient Beta	R square	P value
**Sex (female)**	0.183	0.034	0.392
**Metastatic setting (%)**	0.227	0.052	0.286
**Age (mean)**	-0.314	0.099	0.155
**Follow-up (months)**	-0.175	0.031	0.516

### Disease-free survival

A total of 5 studies comprising 961 patients reported HRs for DFS. Two of these 5 studies (40%) reported a non-statistically significant HR (i.e., the 95% confidence intervals crossed 1). A forest plot of all studies is presented as **Supplementary Figure 2** (available online). Overall, HLA-G expression was associated with DFS which approached but did not meet statistical significance (HR for DFS of 1.74 95% CI = 0.98 to 3.10; P <.06).

In the *post-hoc* sensitivity analysis, 1 study (Du et al, 2011) (21) was excluded as it did not adjust for age in the multivariable model. Again, results showed unchanged effect size (HR 1.69, 95% CI 0.83 to 3.47, p<.15), although with loss of power from a smaller cumulative sample size, confidence intervals were wider.

## Discussion

In this article, we describe the association between expression of HLA-G and clinical outcome in several solid tumors. Given the success for antibody-mediated targeting of inhibitory immunologic signals on adaptive or innate immune cells, such as the targeting of programmed death receptor 1 (PD-1) and its ligand, and of CTLA4; focus has shifted to identify other receptors or ligands with immune inhibitory properties. Several of these targets are under evaluation and some have demonstrated benefit in patients (e.g. LAG3 antibodies in melanoma) (41).

HLA-G is another marker of interest in this setting. The presence of HLA-G is associated with physiological conditions where the immune system is inhibited, such as fetal tolerance during pregnancy (1–5). High expression of HLA-G in solid tumors has also been shown to play an immune-modulatory role in cancer (7, 8). It has been hypothesized that HLA-G works as an immune checkpoint inhibitor in cancer, allowing malignant cells to escape immune cell surveillance (3, 4). Furthermore, it has been shown that HLA-G expression in cancer cell lines can make them less susceptible to immune recognition and elimination (42). Consequently, HLA-G has been suggested as a potential target for therapeutic immune checkpoint inhibition (8, 43, 44).

In our analysis we confirmed data published in individual tumor sites and observed that higher expression of HLA-G is associated with worse survival in solid tumors. In line with this, a recent published meta-analysis covering only tumors in gastrointestinal indications, reported that elevated HLA-G expression was indicative of a poor prognosis and adverse clinicopathological parameters (45). Our study confirms these results adding additional tumor types, and a more represented population of early-stage/curable malignancies. Tumors with higher magnitude association with worse survival included gastric, pancreas and colorectal cancer. While this effect was observed predominantly in tumors with advanced disease, similar effects were observed on studies which included a mixed population of early-stage and advanced-stage patients. Of note the effect was stronger, although not statistically significant, in tumors with early-stage disease. This observation was likely the effect of lower statistical power. Although intra-heterogeneity is associated with a more immunosuppressive environment and less immunoreactivity, we did not observe differences in the prognostic role of HLA-G in early-stage tumors, which are usually more homogeneous, than in metastatic tumors (46, 47). Of note, in one study (Sideras et al., 2017) (32), associations were in the opposite direction of the pooled effect. In this study, positivity was considered as any case with positive staining. This threshold may not have been high enough to identify tumors in which HLA-G expression is meaningful. However, overall, data confirm the importance of this molecule favoring an immune suppressive environment from early stages of disease. Unfortunately, data for DFS were less robust as only 5 studies met the criteria for analysis. With the expectation of lower power in this group, the non-significant association with worse outcome was expected and most likely reflects low sampling in this cohort. Of interest, some studies not included in the current meta-analysis due to lack of reporting of outcome data suggest no association between HLA-G expression and a more aggressive features or adverse risk profile (48, 49).

When evaluating the effect of methods of analysis of HLA-G expression including the antibody used for IHC, or the analysis of the protein in human samples by ELISA, no differences were observed, and all the methods predicted for outcome to a similar magnitude. However, it is important to highlight (see **Supplementary Table 1**) that there was variability in the methods that each study used for analysis, including the cut-off used for consideration of increase HLA-G expression. Of note 56% of the studies utilized the 4H84 clone antibody which represents all α1 domain containing HLA-G or HLA-G5/6 isoforms (7). These data are of interest as they suggest the potential utility of serial liquid biopsies in the evaluation of this biomarker when evaluating therapeutic strategies against this pathway. In this regard, the use of ctDNA has recently demonstrated the potential to stratify patients and predict response to check point inhibitors (50).

Therapeutic strategies targeting HLA-G are currently in clinical development. For instance, MK4830 is an antibody in early phase clinical development targeting the HLA-G receptor, ITL4. In the early phase I study signs of activity were reported in PD-1 pretreated patients (51) Other studies evaluating antibodies targeting ITL2 such as BND22 are in preclinical and early clinical development (12, 52). Early signs of clinical activity have been reported in microsatellite instability-high (MSH-I) colorectal tumors and ovarian cancer, among other tumor types. These observations have supported further clinical exploration (53). In some of these studies, evaluation of HLA-G expression in human samples is explored to assess target inhibition as well as other pharmacodynamic markers.

Our study has limitations. First, this is a meta-analysis of published articles and therefore relies on summary data. Availability of individual patient data would have provided a more accurate estimate of effect. Second, we identified a potential for publication bias in the studies analyzed. Third, there was substantial heterogeneity between studies. While this was expected with the inclusion of diverse solid tumors, attempts to explore the impact of heterogeneity such as subgroup and sensitivity analyses may not have explained the effect of this heterogeneity in full. As such, there remains some uncertainty about effect size estimates. Some subgroups were small in size (e.g. early-stage disease) and therefore there was limited power to explore if magnitudes of effect observed were statistically significant or not. Finally, while all the hazard ratios which we extracted data were derived from multivariable analyses, the variables included in models of individual studies differed. It is for this reason that we elected to further explore variability in the magnitude of the hazard ratio utilizing meta-regression.

In summary, we describe the first meta-analysis evaluating HLA-G expression and its association with worse survival in a variety of solid tumors. This suggests a role of this ligand favoring an immune suppressed environment. Further prospective studies should explore the potential role of HLA-G as a therapeutic target, or as a biomarker of response to agents against the receptors LILRB1, LILRB2 or KIR2DL4.

## Data availability statement

The datasets presented in this study can be found in online repositories. The names of the repository/repositories and accession number(s) can be found in the article/**Supplementary Material**.

## Author contributions

All authors contributed to the article and approved the submitted version.

## Supplementary material

Supplementary Figure 1 Forest plots showing hazard ratio for OS for HLA-G positive. **(A)** Hazard ratios by HLA-G detection type. **(B)** Hazard ratios by HLA-G detection technique. **(C)** Hazard ratios by antibody used. Hazard ratios for each study are represented by the squares, the size of the square represents the weight of the study in the meta-analysis, and the horizontal line crossing the square represents the 95% confidence interval (CI). All statistical tests were two-sided.

Supplementary Figure 2 Forest plot showing hazard ratio for DFS for HLA-G positive. Hazard ratios for each study are represented by the squares, the size of the square represents the weight of the study in the meta-analysis, and the horizontal line crossing the square represents the 95% confidence interval (CI). All statistical tests were two-sided.
