# Supplementary figures and images for: Prognostic value of human leukocyte antigen G expression in solid tumors: a systematic review and meta-analysis
Source: Front Immunol. 2023 May 18;14:1165813. doi: 10.3389/fimmu.2023.1165813 (PMC10232772; doi:10.3389/fimmu.2023.1165813)

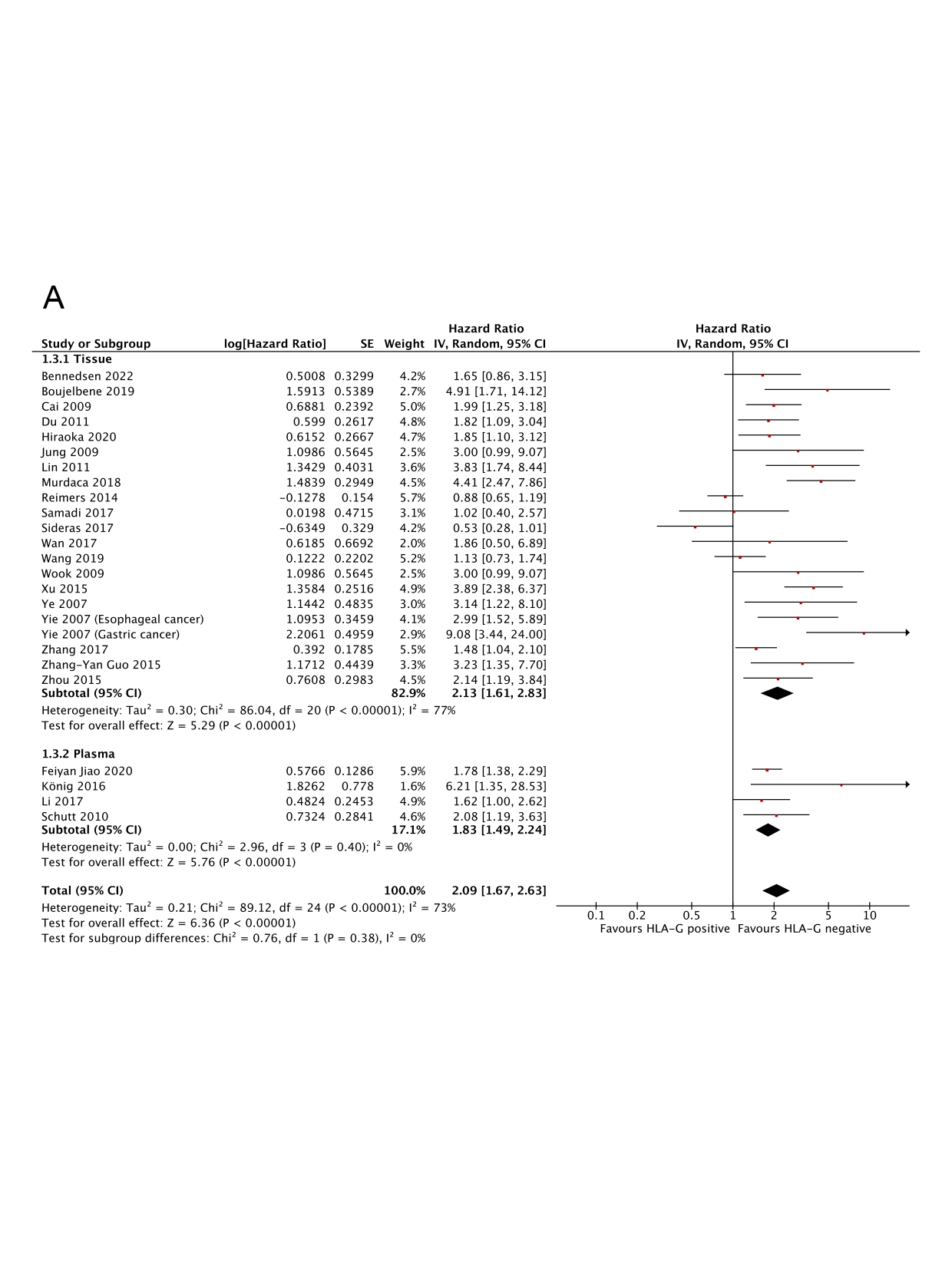

Supplement: Supplementary Figure 1 — Forest plots showing hazard ratio for OS for HLA-G positive. (A) Hazard ratios by HLA-G detection type. (B) Hazard ratios by HLA-G detection technique. (C) Hazard ratios by antibody used. Hazard ratios for each study are represented by the squares, the size of the square represents the weight of the study in the meta-analysis, and the horizontal line crossing the square represents the 95% confidence interval (CI). All statistical tests were two-sided. [file Image_1.jpg]

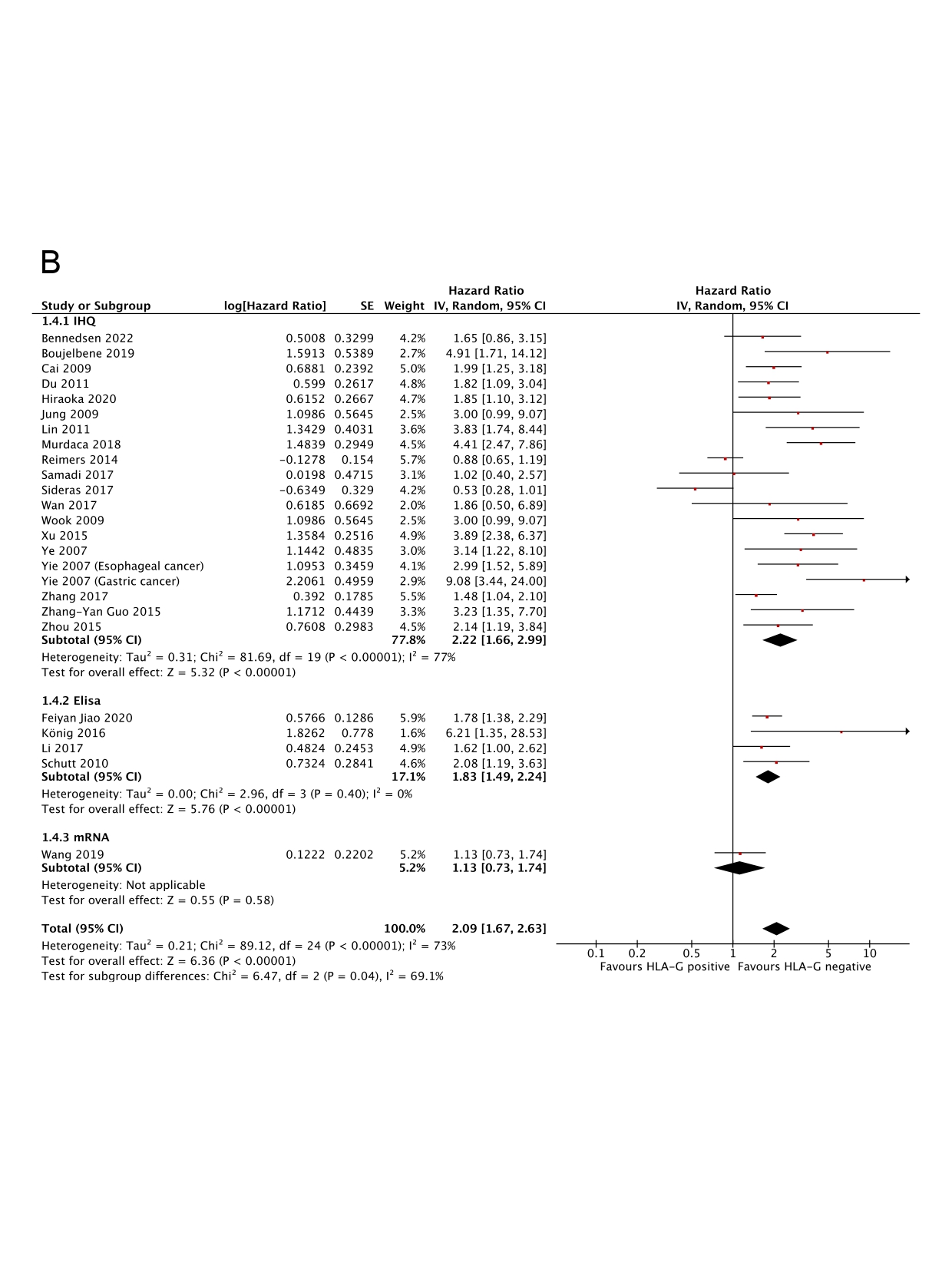

Supplement: Supplementary Figure 2 — Forest plot showing hazard ratio for DFS for HLA-G positive. Hazard ratios for each study are represented by the squares, the size of the square represents the weight of the study in the meta-analysis, and the horizontal line crossing the square represents the 95% confidence interval (CI). All statistical tests were two-sided. [file Image_2.jpg]

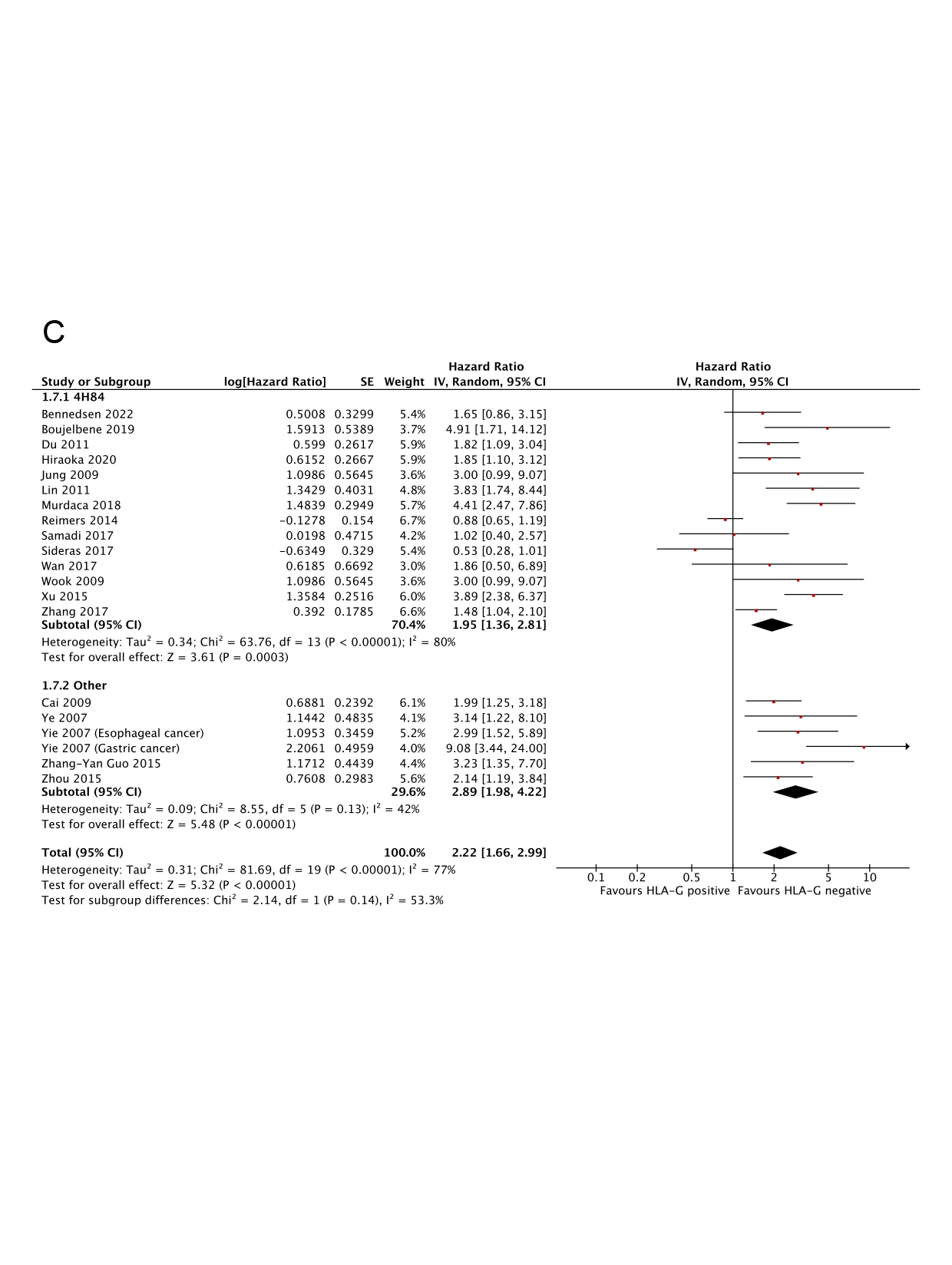

Supplement: Supplementary file 3 [file Image_3.jpg]

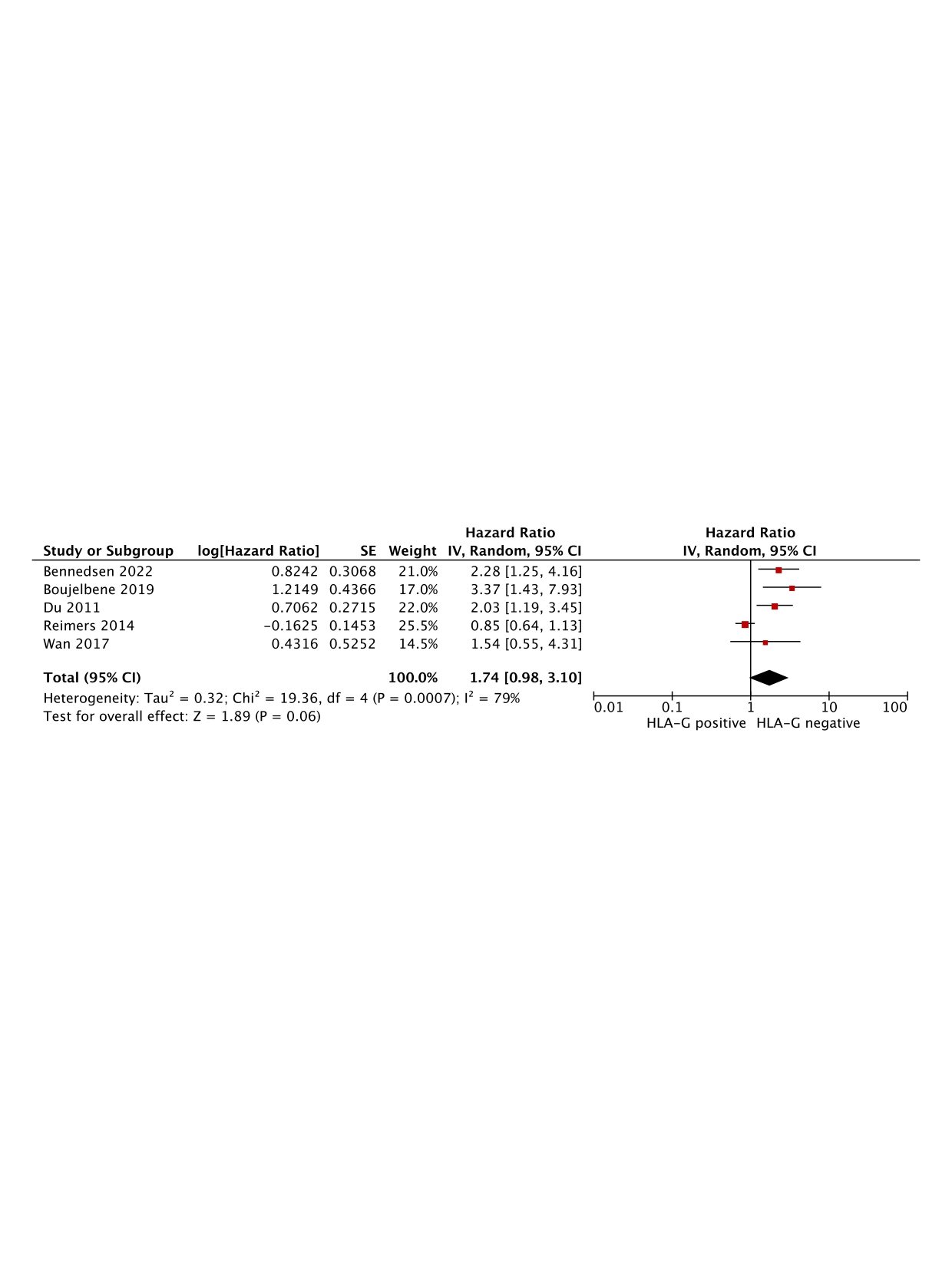

Supplement: Supplementary file 4 [file Image_4.jpg]
