# Supplementary material for: Prognostic value of human leukocyte antigen G expression in solid tumors: a systematic review and meta-analysis
Source: Front Immunol. 2023 May 18;14:1165813. doi: 10.3389/fimmu.2023.1165813 (PMC10232772; doi:10.3389/fimmu.2023.1165813)
Supplement: Supplementary file 5 [file Table_1.docx]

**Supplementary Table 1.**

| **Studies**  **(N = 25)** | **Patients (N)** | **Disease site** | **Histological type**  **N (%)** | **Stage**  **N (%)** | **HLA-G positive definition** | **Reference** |
| --- | --- | --- | --- | --- | --- | --- |
| Bennedsen 2022 | 188 | Colorectal Cancer | Adenocarcinoma NOS, high or moderate diferentiated 124 (66.0)  Adenocarcinoma, poorly diferentiated 32 (17.0)  Mucinous adenocarcinoma 29 (15.4)  Signet ring cell carcinoma 2 (1.1)  Other carcinoma type 1 (0.5) | II 90 (47.9)  III 82 (43.6)  IV 16 (8.5) | ≥10 positive cells per whole slide. A positive cell was defined as cytoplasmic or membrane staining of any intensity. | Bennedsen ALB, et al. An exploration of immunohistochemistry-based prognostic markers in patients undergoing curative resections for colon cancer. *BMC Cancer*. 2022 Jan 14;22(1):62 |
| Boujelbene 2019 | 61 | Vulvar Cancer | Squamous cell carcinoma 61 (100.0) | I 29 (47.5)  II 4 (6.6)  III 26 (42.6)  IV 2 (3.3) | The fraction of stained cells was scored according to the following criteria: score 0 (no stained cells), score 1 (≤25% stained cells), score 2 (≤50% stained cells), score 3 (≤75% stained cells), and score 4 (>75% stained cells). Expression was considered as HLA-G low for score 1. The others expression scores were considered as HLA-G high. | Boujelbene N, et al. HLA-G, HLA-E, and IDO overexpression predicts a worse survival of Tunisian patients with vulvar squamous cell carcinoma. *HLA*. 2019 Jul;94(1):11-24 |
| Cai 2009 | 173 | Hepatocarcinoma | Hepatocellular carcinoma 173 (100.0) | BCLC A 76 (43.9)  BCLC B 45 (26.0)  BCLC C 52 (30.1) | The density of HLA-G – positive staining was evaluated using a computerized image system, including a Leica DFC420charge-coupled device camera and a Leica DM IRE2 microscope (Leica Microsystems Imaging Solution Ltd.). The optimal cut-off for dichotomizing HLA-G expression data was determined using X-tile 3.6.1 software. | Cai MY, et al. Human leukocyte antigen-G protein expression is an unfavorable prognostic predictor of hepatocellular carcinoma following curative resection. *Clin Cancer Res*. 2009 Jul 15;15(14):4686-93. |
| Du 2011 | 179 | Gastric Cancer | NA | I 40 (22.3)  II 34 (19.0)  III 40 (22.3)  IV 65 (36.3) | HLA-G was interpreted as positive or negative and the immunoreactivity was  further graded as follows by the percentage of positively stained cancer cells: 0%; +, 1–25%; ++, 26–50%; and +++, >50%. | Du L, et al. Human leukocyte antigen-G is closely associated with tumor immune escape in gastric cancer by increasing local regulatory T cells. *Cancer Sci.* 2011 Jul;102(7):1272-80. |
| Feiyan Jiao 2020 | 1037 | Colorectal Cancer | NA | I 144 (13.9)  II 337 (32.5)  III 519 (50.0)  IV 37 (3.6) | According to the cut-off value, the patients were divided into high-level group (sHLA-G ≥50.8 U/mL) and low-level group (sHLA-G <50.8 U/mL). | Jiao F, et al. Plasma soluble human leukocyte antigen G predicts the long-term prognosis in patients with colorectal cancer. *Transl Cancer Res.* 2020 Jun;9(6):4011-4019. |
| Hiraoka 2020 | 98 | Pancreatic Cancer | Pancreatic ductal adenocarcinoma 98 (100) | M0 88 (89.8)  M1 10 (10.2) | When more than 5% of cancer cells in a PDAC tissue section expressed HLA-G, the PDAC case was judged positive for HLA-G; otherwise, it was considered negative. | Hiraoka N, et al. Expression of classical human leukocyte antigen class I antigens, HLA-E and HLA-G, is adversely prognostic in pancreatic cancer patients. *Cancer Sci.* 2020 Aug;111(8):3057-3070. |
| Jung 2009 | 41 | Ovarian Cancer | Papillary serous cell carcinoma 25 (60.9)  Mucinous cell carcinoma 4 (9.8)  Endometrioid carcinoma 4 (9.8)  Clear cell carcinoma 6 (14.6)  Undifferentiated carcinoma 2 (4.9) | I 7 (17.1)  II 5 (12.2)  III 24 (58.5)  IV 5 (12.2) | > 17% HLA-G staining. | Jung YW, et al. Correlation of human leukocyte antigen-G (HLA-G) expression and disease progression in epithelial ovarian cancer. *Reprod Sci.* 2009 Nov;16(11):1103-11. |
| König 2016 | 190 | Breast Cancer | Ductal 140 (73.7)  Lobular 22 (11.6)  Other 24 (12.6)  Unknown 4 (2.1) | Non-metastatic 190 (100) | sHLA-GEV plasma levels greater than 15 ng/ml. | König L, et al. The prognostic impact of soluble and vesicular HLA-G and its relationship to circulating tumor cells in neoadjuvant treated breast cancer patients. *Hum Immunol.* 2016 Sep;77(9):791-9. |
| Li 2017 | 178 | Colorectal Cancer | NA | I 24 (13.5)  II 54 (30.3)  III 85 (47.8)  IV 6 (3.4) | sHLA-G high above the median of 36.8 U/ml. | Li JB, Ruan YY, Hu B, Dong SS, Bi TN, Lin A, Yan WH. Importance of the plasma soluble HLA-G levels for prognostic stratification with traditional prognosticators in colorectal cancer. *Oncotarget*. 2017 Jul 25;8(30):48854-48862. |
| Lin 2011 | 79 | Esophageal Cancer | Esophageal squamous cell carcinoma 79 (100) | I 3 (3.8)  II 35 (44.3)  III 37 (46.8)  IV 4 (5.1) | HLA-G expression was graded as follows: negative, 1–25% (1+), 26–50% (2+), 51–  75% (3+) and >75% (4+). | Lin A, Zhang X, Zhou WJ, Ruan YY, Xu DP, Wang Q, Yan WH. Human leukocyte antigen-G expression is associated with a poor prognosis in patients with esophageal squamous cell carcinoma. *Int J Cancer*. 2011 Sep 15;129(6):1382-90. |
| Murdaca 2018 | 94 | Gastric Cancer | Tubular 71 (75.5)  Signet cell 11 (11.7)  Mucinous 7 (7.4)  Papillary 5 (5.3) | I 14 (14.9)  II 40 (42.6)  III 40 (42.6) | Semi-quantitative assessment of HLA-G staining  intensity was performed and reported as follows: strong ex-  pression as in normal placenta vs weak expression (i.e., less intense than normal placenta) vs absence of staining. | Murdaca G, et al. HLA-G expression in gastric carcinoma: clinicopathological correlations and prognostic impact. *Virchows Arch.* 2018 Oct;473(4):425-433. |
| Reimers 2014 | 484 | Colorectal Cancer | NA | I 134 (27.1)  II 136 (27.5)  III 193 (39.0)  IV 32 (6.5) | For analysis were categorized as weak (absent and weak intensity together) versus strong (moderate and strong intensity together) tumor staining. | Reimers MS, et al. Prognostic value of HLA class I, HLA-E, HLA-G and Tregs in rectal cancer: a retrospective cohort study. *BMC Cancer*. 2014 Jul 5;14:486. |
| Samadi 2017 | 100 | Colorectal Cancer | NA | I 14 (14)  II 45 (45)  III 37 (37)  IV 4 (4) | Positive HLA-G expression. | Samadi R, et al. Clinical Value of Human Leucocyte Antigen G (HLA-G) Expression in the Prognosis of Colorectal Cancer. *Int J Cancer Manag*. 2017 April; 10(4):e9346. |
| Schutt 2010 | 137 | Lung Cancer | SCLC 23 (17)  NSCLC 114 (83)  Adenocarcinoma 55 (41)  SCC 46 (33)  Undifferentiated carcinoma 13 (9) | I/II 37 (27)  III 52 (38)  IV 48 (35) | sHLA-G cut-off 40 ng/ml. | Schütt P, et al. Prognostic relevance of soluble human leukocyte antigen-G and total human leukocyte antigen class I molecules in lung cancer patients. *Hum Immunol.* 2010 May;71(5):489-95. |
| Sideras 2017 | 224 | Pancreatic Cancer | Pancreatic cancer 148 (66.1)  Ampullary cancer 76 (33.9) | NA | Any case with positive staining was considered high and all negative cases were considered low. | Sideras K, et al. Tumor cell expression of immune inhibitory molecules and tumor-infiltrating lymphocyte count predict cancer-specific survival in pancreatic and ampullary cancer. *Int J Cancer.* 2017 Aug 1;141(3):572-582. |
| Wan 2017 | 49 | Gastric Cancer | Adenocarcinoma 44 (89.8)  Other 5 (10.2) | I 20 (40.8)  II 23 (46.9)  III 4 (8.1)  IV 2 (4.1) | >10% HLA-G positive cells. | Wan R, et al. Human Leukocyte Antigen-G Inhibits the Anti-Tumor Effect of Natural Killer Cells via Immunoglobulin-Like Transcript 2 in Gastric Cancer. *Cell Physiol Biochem*. 2017;44(5):1828-1841. |
| Wang 2019 | 212 | Hepatocarcinoma | NA | BCLC 0 20 (9.4)  BCLC A 143 (67.5)  BCLC B 22 (10.2)  BCLC C 27 (12.7) | High expression. | Wang XK, et al. Diagnostic and prognostic biomarkers of Human Leukocyte Antigen complex for hepatitis B virus-related hepatocellular carcinoma. *J Cancer.* 2019 Aug 28;10(21):5173-5190. |
| Wook 2009 | 41 | Ovarian Cancer | Other type 33 (80.5)  Clear and undifferentiated 8 (19.5) | I-II 12 (29.3)  III-IV 29 (70.7) | >17 HLA-G staining. | Jung YW, et al. Correlation of human leukocyte antigen-G (HLA-G) expression and disease progression in epithelial ovarian cancer*. Reprod Sci.* 2009 Nov;16(11):1103-11. |
| Xu 2015 | 122 | Pancreatic Cancer | Pancreatic ductal adenocarcinoma 122 (100) | I 13 (10.6)  II 109 (89.4) | The proportion score was calculated by the proportion of positive staining of tumor cells (0, none; 1, <=25%; 2, 26-50%; 3, >50%). The intensity  score measures staining intensity from a range of 0 to  3 (0, none; 1, weak; 2, moderate; 3, strong). The score  of expression of HLA-G was a comprehensive evaluation score according to positive incidence and intensity grading. | Xu YF, et al. High Expression of Human Leukocyte Antigen-G is Associated with a Poor Prognosis in Patients with PDAC. *Curr Mol Med.* 2015;15(4):360-7. |
| Ye 2007 | 201 | Colorectal Cancer | Adenocarcinoma 201 (100) | I 42 (21)  II 77 (38)  III 78 (39)  IV 4 (2) | Negative: tissue specimens without staining.  Positive:  (+) tissue specimens with less  than 25% of the cancer tissues and/or weakly stained,  (++) tissue specimens with 25–50% of the cancer tissue and/or moderately stained and  (+++) tissue specimens with more than 50% of the cancer tissue and/or strongly stained. | Ye SR, et al. Human leukocyte antigen G expression: as a significant prognostic indicator for patients with colorectal cancer*. Mod Pathol.* 2007 Mar;20(3):375-83. |
| Yie 2007 | 121 | Esophageal Cancer | Esophageal squamous cell carcinoma 121 (100.0) | I 16 (13.2)  II 36 (29.8)  III 53 (43.8)  IV 16 (13.2) | Negative, no staining.  1+, <25% of specimen is cancer and stained weakly.  2+, 25%-50% of specimen is cancer and stained moderately.  3+, >50% of specimen is cancer and stained strongly. | Yie SM, et al. Expression of HLA-G is associated with prognosis in esophageal squamous cell carcinoma. *Am J Clin Pathol.* 2007 Dec;128(6):1002-9. |
| Yie 2007 | 160 | Gastric Cancer | Adenocarcinoma 138 (86.3)  Signet cell carcinoma 13 (8.1)  Adenosquamous carcinoma 9 (5.7) | Ia 11 (7.0)  Ib 59 (37.0)  II 59 (37.0)  IIIa 37 (23)  IIIb 12 (7.0)  IV 10 (6.0) | (–) tissue specimens without staining.  (+) tissue specimens with less than 25% of the cancer tissues and/or weakly stained.  (++) tissue specimens with 25–50% of the cancer tissues and/or moderately stained.  (+++) tissue specimens with more than 50% of the cancer tissues and/or strongly stained. | Yie SM, et al. Expression of human leukocyte antigen G (HLA-G) correlates with poor prognosis in gastric carcinoma. *Ann Surg Oncol*. 2007 Oct;14(10):2721-9. |
| Zhang 2017 | 457 | Colorectal Cancer | NA | I 90 (19.7)  II 149 (32.6)  III 200 (43.8)  IV 16 (3.5) | HLA-G low cut-off 5%  HLA-G high cut-off 55% | Zhang RL, et al. Predictive value of different proportion of lesion HLA-G expression in colorectal cancer. *Oncotarget.* 2017 Nov 18;8(64):107441-107451. |
| Zhang-Yan Guo 2015 | 102 | Colorectal Cancer | Tubular adenocarcinoma 6 (5.9)  Adenocarcinoma 85 (83.3)  Ulcertypetubularadenocarcinoma 1 (0.98)  Mucinous adenocarcinoma 10 (9.8) | M0 96 (94.1) M1 6 (5.9) | Positive expression of HLA-G in the membrane and/or the cytoplasm. | Guo ZY, et al. Predictive value of HLA-G and HLA-E in the prognosis of colorectal cancer patients. *Cell Immunol.* 2015 Jan;293(1):10-6. |
| Zhou 2015 | 143 | Pancreatic Cancer | NA | Non-metastatic 143 (100) | HLA-G-positive cells was classified into three grades (Negative: <5%; local: 5-75%;  diffuse: >75%), whereas its staining intensity was not considered. | Zhou L, et al. HLA-G impairs host immune response and predicts poor prognosis in pancreatic cancer. *Am J Transl Res*. 2015 Oct 15;7(10):2036-44. |

NA = Not available.
